# Design and Experimental Validation of a Dynamic Frequency Sweeping Algorithm for Optimized Impedance Matching in Semiconductor RF Power Systems Under Pulse-Mode Operation

**DOI:** 10.3390/mi17030376

**Published:** 2026-03-20

**Authors:** Zhaolong Fan, Zhifeng Wang, Long Xu, Lili Hou, Long Yao, Siao Zeng, Mingqing Liu

**Affiliations:** 1 Jihua Laboratory, Foshan 528200, China; fanzl@jihualab.ac.cn (Z.F.); xulong@jihualab.com (L.X.); houll@jihualab.com (L.H.); 2 Guangdong Provincial Key Laboratory of Industrial Intelligent Inspection Technology, Foshan University, Foshan 528000, China; zhifengw0816@163.com (Z.W.); siaoz0717@163.com (S.Z.); 2112551036@stu.fosu.edu.cn (M.L.); 3 School of Electromechanical Engineering, Guangdong University of Technology, Guangzhou 510006, China

**Keywords:** dynamic frequency sweeping, impedance matching, pulse-mode operation, power transfer efficiency, adaptive algorithm

## Abstract

The design and implementation of a dynamic frequency sweeping algorithm for a 3 kW RF power source are underpinned by theoretical principles aimed at optimizing impedance matching under pulse-mode operation. The algorithm dynamically adjusts the output frequency within a predefined range to align the source impedance *Z_source_* with the conjugate of the load impedance Z*_load_, maximizing the power transfer efficiency and minimizing the reflection coefficient *Γ*. This is achieved by leveraging the maximum power transfer theorem and adapting to dynamic load variations, such as those induced by the plasma state transitions. The algorithm incorporates adaptive step size adjustments based on the rate of change of *Γ*, predictive frequency initialization using historical data, and real-time impedance monitoring to ensure efficient convergence within the constrained pulse “*ON*” time (*TON*). Integration with pulse mode requires synchronization with the pulse signal, fast convergence, and optimized search strategies. Experimental validation on a 13.56 MHz, 3 kW Automatic Sweep Generator testbed operating at 20 kHz pulse modulation with a 50% duty cycle demonstrates a linear and stable sweep, achieving impedance matching and low reflected power within 5.0172 ms. These findings highlight the algorithm’s potential for high-precision applications, such as RF plasma excitation, and underscore the importance of adaptive techniques in dynamic RF systems.

## 1. Introduction

**Figure 1:**
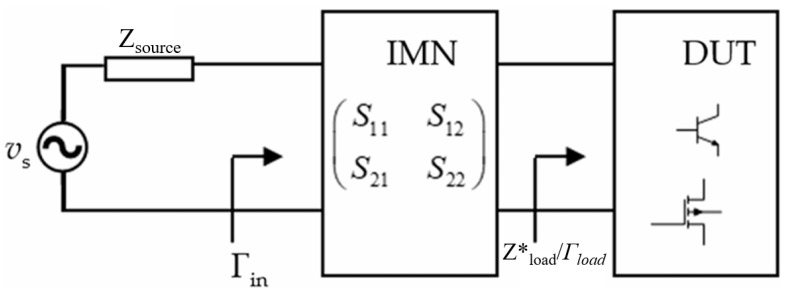
Matching an arbitrary load impedance Z_L_ to a source impedance Z_S_.

The primary role of the RF power supply is to excite plasma by providing high-frequency alternating current power (typical frequencies of 13.56 MHz or 27.12 MHz), thereby enabling key semiconductor wafer processes such as surface modification, etching, and deposition. Capacitively coupled plasmas (CCPs) are widely utilized in industrial applications such as material processing, thin film deposition, and plasma etching, owing to their ability to generate uniform plasma over large areas [1,2,3,4,5,6,7]. Efficient power transfer from the radio frequency (RF) source to the plasma is crucial for optimizing the process performance, typically achieved through an impedance matching network (IMN) that aligns the complex plasma impedance with the characteristic impedance of the power delivery system, often 50 Ω [8,9]. However, dynamic load variations, such as those induced by plasma state transitions, gas pressure changes, or thermal fluctuations, challenge traditional impedance matching approaches, necessitating advanced techniques [10,11,12]. To match a source impedance *Zsource* to an arbitrary load impedance Z*load, as illustrated in Figure 1, various theories and techniques have been developed. To meet this objective, Fano [13] and Youla [14] invoked the gain-bandwidth limitation theory and utilized the Dar lington representation, where the load Z*load is represented by a lossless two-port terminated in a unit resistor. Analytical models for determining the IMN are also used, but they require an analytical model of the load impedance [15] and often result in suboptimal solutions in terms of circuit performance metrics and network complexity [16]. Another approach to the design of passive IMNs relies on filter theory [17,18,19,20]. The approach employs either a cascaded L-section topology consisting of high-pass and low-pass filters [17,18] or a bandpass filter [19,20]. These fixed topologies, however, may not necessarily provide a minimum number of elements in the IMN for a specific load [16]. A related approach employs series or tank resonators, and it is based on manipulations of the resonant frequencies [21,22]. When these resonant frequencies can be strategically spaced apart while maintaining an acceptable gain variation, it is possible to attain a broadband IMN. However, it may not prove effective for high fractional bandwidth (FBW) requirements, where many resonators are required. At the other way, studies have explored impedance matching for single-frequency [23,24], dual-frequency [25,26], and multi-frequency CCPs [27,28,29], yet few have addressed the impact of coaxial cables, which act as transmission lines (TLs) and significantly influence voltage, current, and impedance matching when their length is comparable to the signal wavelength [30,31,32,33]. The integration of RF coaxial cables in CCP systems introduces distributed circuit effects that complicate impedance matching design [33]. Experimental evidence, such as that from Du et al., highlights the cables’ influence on DC self-bias and harmonic components [31], underscoring the need for comprehensive models that couple TLs, lumped circuits, and plasma dynamics [32]. The TLM is directly solved by the Lax–Wendroff method (LWM) and bidirectionally coupled with a lumped-element circuit model of the matching network and an electrostatic PIC model of the plasma [34,35]. The optimal SRFT solution requires fewer components in the impedance matching network and allows for circuit optimization to reduce power consumption [36]. While prior works have coupled TL models with particle-in-cell/Monte-Carlo collision (PIC/MCC) simulations [37], a systematic investigation of cable effects on impedance matching remains limited. This gap motivates the development of adaptive algorithms to dynamically adjust the RF source frequency, ensuring optimal power delivery under pulse-mode operation.

This paper proposes a dynamic frequency sweeping algorithm for a 13.56 MHz RF. power source, designed to achieve conjugate impedance matching (*Zsource* = Z*load) and minimize the reflection coefficient Γ by leveraging the maximum power transfer theorem. The algorithm incorporates adaptive step size adjustments, predictive frequency initialization, and real-time impedance monitoring to ensure fast convergence within the pulse “*ON*” time (*TON*). Experimental validation on a 13.56 MHz, 3 kW Automatic Sweep Generator operating at 20 kHz with a 50% duty cycle demonstrates a stable sweep, achieving impedance matching and low reflected power within 5.0172 ms. This approach enhances the precision of RF plasma excitation and offers a robust solution for dynamic RF systems. The paper is organized as follows: Section 2 details the theoretical foundations and algorithm design, Section 3 describes the experimental setup and results, and Section 4 concludes with key findings and future directions.

## 2. Theoretical Foundations Underpinning the Algorithm Design

The proposed dynamic frequency sweeping algorithm is specifically designed for pulse-mode operation in 13.56 MHz RF power systems. It dynamically adjusts the output frequency within a predefined range to track the time-varying resonant frequency of the load in real time, thereby achieving conjugate impedance matching (*Z_source_* = Z*_load_) and minimizing the reflection coefficient Γ.

Conventional fixed-step frequency sweeping is inefficient in pulse-mode applications due to the extremely short pulse “ON” time (*T_ON_* typically 10–50 μs), which severely limits the number of evaluable frequency points. To overcome this limitation, the proposed algorithm introduces a gradient-based adaptive step size mechanism:

(1) $$\Delta f=\frac{k}{\left|\frac{d\Gamma }{df}\right|}$$

where k is a tunable scaling factor (empirically set to 10^4^), when far from resonance ($\left|\frac{d\Gamma }{df}\right|$ is small), a larger step size is employed to accelerate global search. When near resonance ($\left|\frac{d\Gamma }{df}\right|$ is large), the step size is automatically reduced to enhance local precision and prevent overshooting.

This mechanism ensures fast and high-precision convergence within the constrained T_ON_. Theoretical analysis demonstrates that the convergence time scales as O(1/max$\left|\frac{d\Gamma }{df}\right|$), offering approximately 60% speedup compared to fixed-step methods with the same initial step size.

Furthermore, to further reduce the number of scan iterations, the algorithm adopts a predictive initialization strategy: the optimal frequency *f*_opt_ from the previous pulse cycle is used as the initial frequency *f*_init_ for the current cycle, restricting the search to a narrow window [*f*_opt_ − Δ*f*_range_, *f*_opt_ + Δ*f*_range_]. Real-time impedance monitoring is performed at each frequency point to update Γ. The algorithm terminates when Γ < Γ_threshold_ (typically 0.05) or when T_ON_ expires, returning the frequency corresponding to the lowest recorded Γ.

These innovations enable the proposed algorithm to significantly outperform conventional methods in convergence speed, robustness, and computational efficiency, making it particularly well-suited for high-precision RF plasma applications under pulse-mode constraints.

## 3. Principles of Integrating Frequency Sweeping with Pulse Mode

The goal of dynamic frequency sweeping is to adjust the output frequency *f* to achieve conjugate impedance matching (Z_source_ = Z*_load_), minimizing the reflection coefficient *Γ.* In pulse mode, the sweeping algorithm must optimize the frequency within each *T_ON_* phase. The integration principles are detailed below:

Frequency Dependence: The reactance component of the load impedance *X_load_* is frequency-dependent:

(2) $$X_{load\;}=2\pi fL-\frac{1}{2\pi fC}$$

where *L* and *C* are the equivalent inductance and capacitance of the load, respectively. When *X_load_* ≈ 0, the system is at resonance, minimizing *Γ*. In pulse mode, *L* and *C* may vary due to plasma state changes, requiring the algorithm to quickly identify the new resonant frequency.

Time Constraints: The sweeping algorithm operates within the *TON* window (typically 10 μs to 10 ms), necessitating:

• Fast Convergence: Finding the optimal frequency within a limited time.
• Efficient Search: Reducing the number of frequency points scanned through optimized step sizes or predictive methods.
• Real-Time Monitoring: Using high-precision sensors (e.g., directional couplers) to measure Γ or reflected power in real time.

Pulse Synchronization: The sweeping algorithm must synchronize with the pulse signal:

• Trigger Mechanism: Initiate sweeping at the start of each “*ON*” phase.
• Frequency Retention: Store the optimal frequency f_opt_ from the previous cycle for use as the initial frequency in the next cycle.
• Dynamic Adjustment: Adjust the sweep range and step size based on the length of T_ON_.

## 4. Algorithm Design for Dynamic Frequency Sweeping in Pulse Mode

To address the challenges of pulse mode, the dynamic sweeping algorithm incorporates the following strategies:

Adaptive Step Size Mechanism: Traditional fixed-step sweeping is inefficient in pulse mode due to the short *T_ON_* window, which limits the number of frequency points that can be scanned. An adaptive step size algorithm adjusts the frequency step Δ*f* based on the rate of change of the reflection coefficient:

(3) $$\Delta f=K{\left|\frac{d\Gamma }{df}\right|}^{-1}$$

• Principle: When $\frac{d\Gamma }{df}$ is large (near the resonant frequency), reduce *Δf* for higher precision; when $\frac{d\Gamma }{df}$ is small (far from resonance), increase *Δf* to speed up the search.
• Advantage: Reduces the number of scanned points within *T_ON_*, improving efficiency.

Predictive Frequency Initialization: To further reduce sweep time, historical data can be used for frequency prediction:

• Record Historical Data: Store the optimal frequency *f*_opt_ from previous pulse cycles.
• Initial Frequency Selection: Start the new *T_ON_* phase with *f_opt_* or a nearby frequency, narrowing the sweep range to [*f_opt_* − *Δfrange*, *fopt* + *Δf_range_*].
• Machine Learning Assistance: For periodic or predictable load changes, machine learning models (e.g., neural networks) can predict *f_opt_* trends.

Real-Time Impedance Monitoring: High-precision sensors (e.g., vector network analyzers or reflected power sensors) measure *Z_source_* or *Γ* in real time. Within each *T_ON_* phase, the algorithm performs:

1. Initialize frequency *f = f*_init_ (typically the previous cycle’s *f_opt_*)
2. Measure current *Γ* and *Z_load_*.
3. Update step size *Δ f* based on $\frac{d\Gamma }{df}$, and adjust frequency to *f* + Δ*f*.
4. If *Γ* < *Γ*_threshold_ (e.g., 0.05), lock the current frequency; otherwise, continue sweeping.

**Figure 2:**
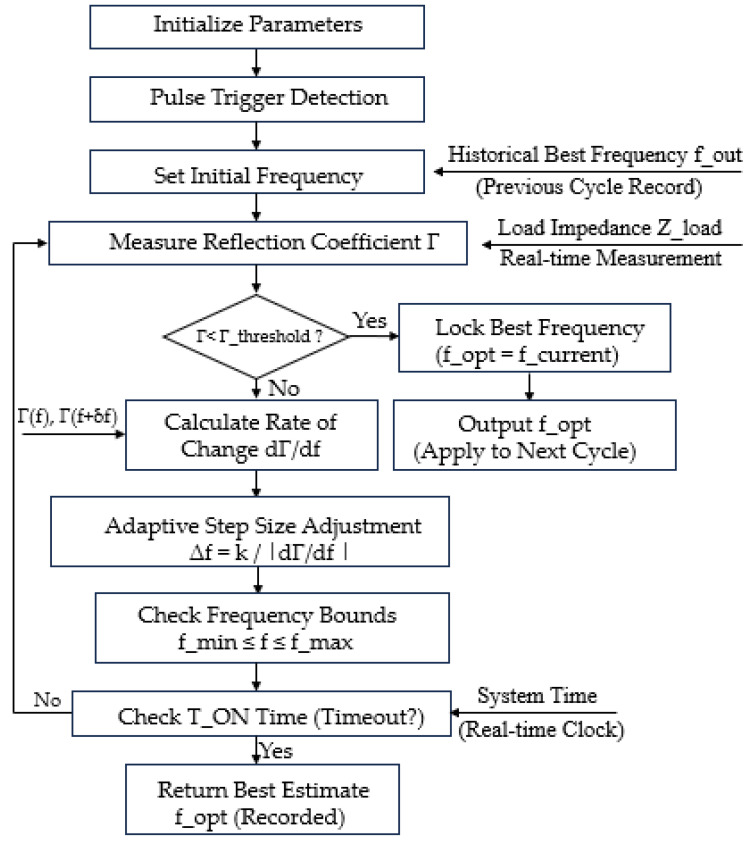
Flowchart of the proposed algorithm.

Algorithm Implementation and Workflow: The following Figure 2 is the Algorithm flow chart for the dynamic sweeping algorithm in pulse mode:

The algorithm is implemented in twelve modular steps, as Algorithm 1 described below:
**Algorithm 1:** Adaptive Dynamic Frequency Sweeping Algorithm.Step 1: Parameter Initialization Initial parameters essential for the algorithm’s operation are configured, including the frequency sweep range [*f_min_, f_max_*] (e.g., 13.06–14.06 MHz), pulse “*ON*” duration *T_ON_* (e.g., 25 µs), initial frequency step size *Δf_0_* (e.g., 10 kHz), reflection coefficient threshold *Γ*_threshold_ (e.g., 0.05), and the initial frequency finit, which is typically set to the previously recorded optimal frequency *f_opt_* or the midpoint of the sweep range. Step 2: Pulse Trigger Detection The start of the pulse “*ON*” phase is detected using either a system clock or an external control signal. Upon detection, a trigger signal initiates the frequency sweep process. Step 3: Initial Frequency Setup The current frequency fcurrent is initialized to *f_init_*, with a preference for using the previous optimal frequency *f_opt_*, if available, to accelerate convergence. Step 4: Reflection Coefficient Measurement The load impedance *Z_load_* is measured at fcurrent using a directional coupler or vector network analyzer. The corresponding reflection coefficient *Γ* is then computed using standard impedance transformation equations. Step 5: Matching Check The algorithm evaluates whether the computed reflection coefficient satisfies the condition *Γ* < *Γ_threshold_*. If the criterion is met, it proceeds to frequency locking; otherwise, the frequency is adjusted. Step 6: Frequency Locking When impedance matching is achieved, the current frequency *f_current_* is recorded as the optimal frequency *f_opt_*, which can be used in the current or subsequent pulse cycle. Step 7: Rate of Change Calculation The variation rate of the reflection coefficient is calculated between adjacent frequencies (e.g., *f_current_* and *f_current_ + δf*), enabling an assessment of local gradient behavior in the impedance matching landscape. Step 8: Adaptive Step Size Adjustment The frequency step size *Δf* is adaptively updated according to the computed rate of change, scaled by a tunable factor K. A maximum allowable step size is enforced to prevent excessive jumps that could destabilize convergence. Step 9: Frequency Update The next frequency point is determined by updating *f_current_ = f_current_ + Δf* Step 10: Frequency Boundary Check To ensure the frequency remains within bounds, the algorithm enforces:•If *f_current_* > *f_max_*, reset *f*_curre_*_n_t* = *f_min_*;•If *f_current_* < *f_min_*, enforce *f_current_* = *f_min_*.Step 11: Pulse Duration Check The algorithm monitors the elapsed time during the “*ON*” period *TON*, the algorithm terminates and returns the current best estimate *f_opt_*. Step 12: Final Frequency Output If no frequency satisfies *Γ* < *Γ_threshold_* within the allowed pulse duration, the frequency associated with the minimum recorded *Γ* is returned as the final estimate *f_opt_*.

## 5. Experimental Results and Analysis

Experimental Platform: The experimental work was carried out on a remote plasma source (RPS) system platform. A custom-designed 13.56 MHz, 3 kW radio frequency (RF) generator was employed as the primary RF source. Signal acquisition and waveform analysis were performed using a Tektronix digital oscilloscope with a bandwidth of at least 500 MHz and dual-channel capability.

Probe Channel 1 was connected to the output port of the RF generator to monitor the forward signal, while Probe Channel 2 was configured to capture the reflected signal. The system was terminated with an RPS load, simulating practical plasma processing conditions. Argon gas was utilized as the ionization medium due to its inert characteristics and common usage in low-pressure plasma applications.

**Figure 3:**
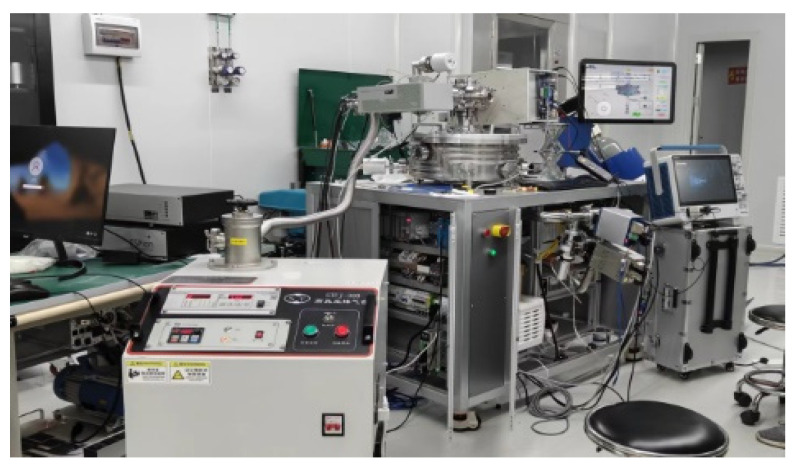

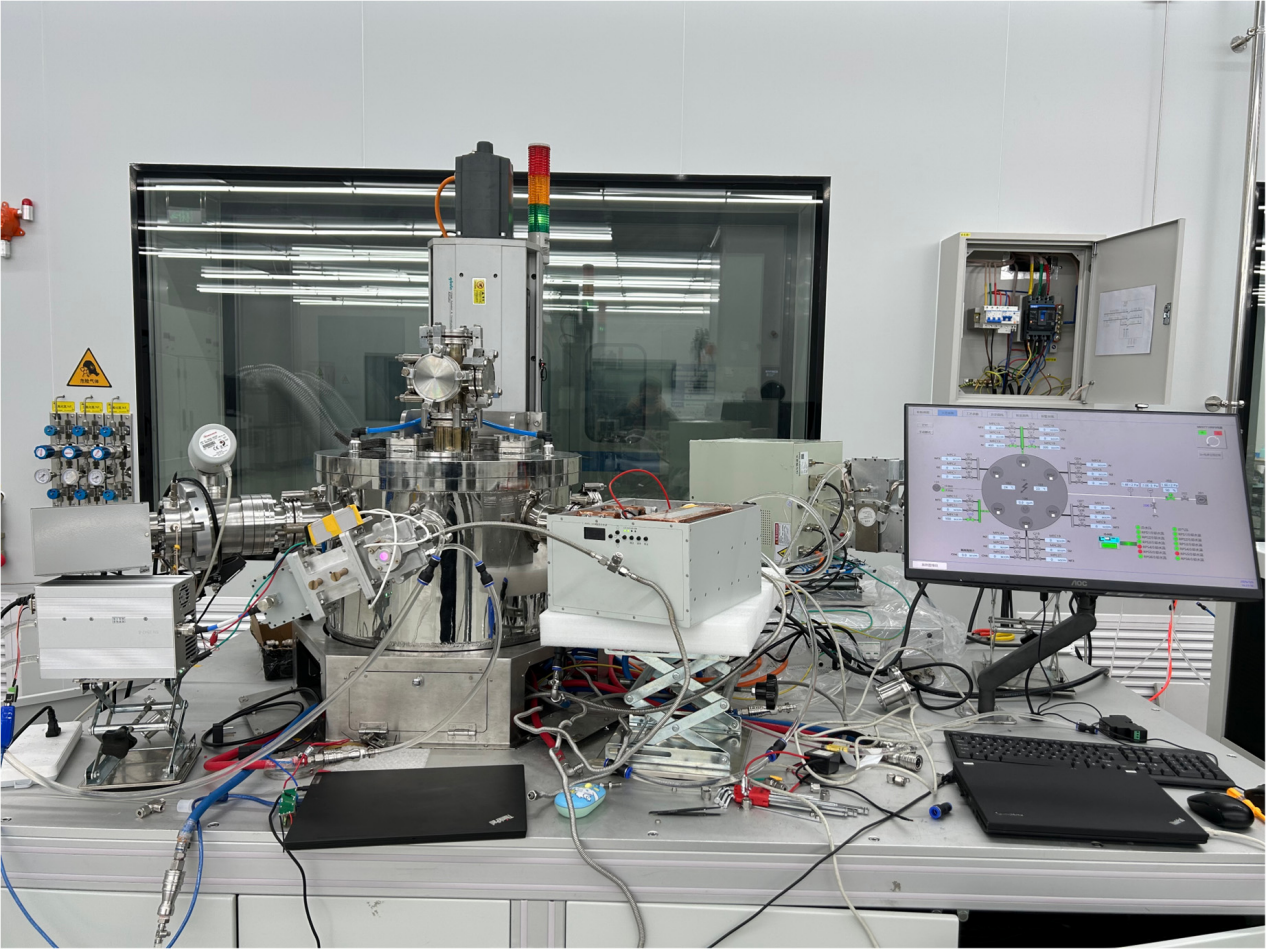
Remote plasma source (RPS) system platform.

The complete experimental hardware configuration is depicted in Figure 3.

The algorithm was implemented in C/C++ language. The configuration of experimental parameters is summarized in Table 1.

For samples 3–5, the Al content of emitter was decreased from 19% to 10% successively, while the other parameters remained the same. Finally, we introduce a double hetero-junction into sample 6.

Experimental Procedure: (a) The vacuum pump and gas valves on the remote plasma source (RPS) system platform were activated, and the chamber was evacuated to a pressure below 30 Pa. (b) Argon gas was introduced into the system until the chamber and internal flow channels were fully filled. (c) The 3 kW RF generator was then started, initiating plasma discharge inside the RPS chamber. (d) The output waveforms were observed and recorded via a digital oscilloscope.

Experimental Results: The RF generator operated in a self-adaptive frequency sweep mode under a 20 kHz pulse modulation. During the sweep, the variations in output signal amplitude and frequency, as well as the corresponding response at the load side, were investigated.

**Figure 4:**
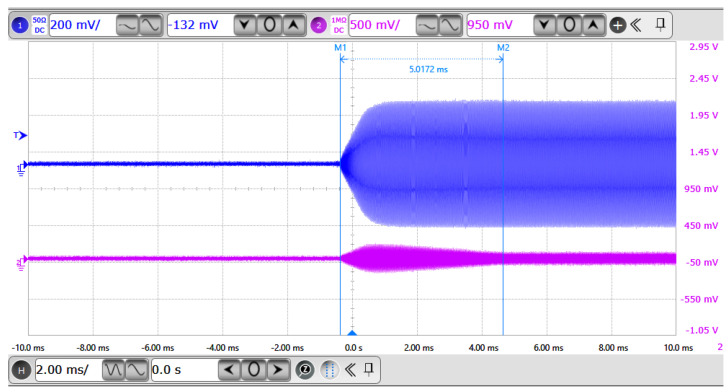
Experimental frequency sweep process of the 13.56 MHz, 3 kW automatic sweep generator.

**Figure 5:**
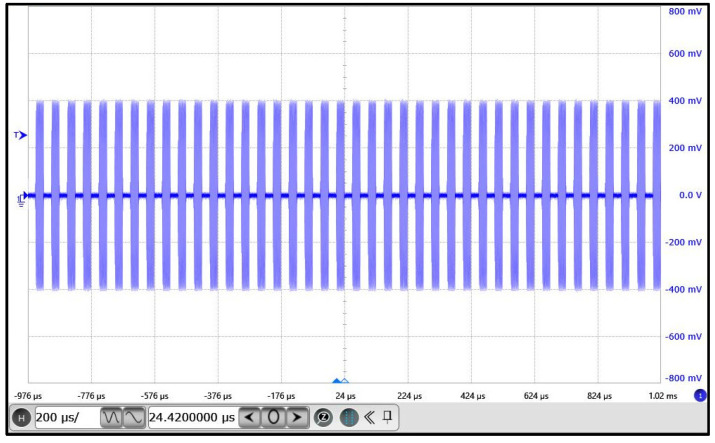
Pulse function of the 13.56 MHz, 3 kW automatic sweep generator during the experiment.

The waveforms of both the RF output and the reflected signal were captured using the oscilloscope. Additionally, the pulse-modulated output behavior of the RF generator was monitored. The experimental results are illustrated in the figures below:

• Figure 4 displays the frequency sweep characteristics: the blue waveform represents the RF output signal, and the purple waveform corresponds to the reflected signal.
• Figure 5 shows the pulse-modulated output of the RF generator under 20 kHz control, where the blue waveform indicates the periodic RF bursts.

Experimental Result Analysis: As shown in Figure 4, the waveform evolution throughout the frequency sweep process can be clearly divided into the following phases:

• Before the sweep (*t* < 0 ms): Both channels display static or DC-level signals, indicating that the RF power source has not yet initiated excitation.
• Sweep initiation (*t* = 0 ms): The blue waveform begins to output a gradually increasing RF signal, both in amplitude and frequency, signifying the onset of frequency sweep.
• During the sweep (0–5 ms): The blue waveform exhibits increasing frequency and waveform density, characteristic of a linear frequency-modulated continuous wave (*LFM-CW*) sweep. The purple waveform (reflected signal) also responds accordingly, suggesting impedance mismatch dynamics during the sweep.
• After the sweep (*t* > 5 ms): The blue waveform stabilizes in both amplitude and frequency, indicating that the frequency sweep has completed and the generator has transitioned into steady-state output. Simultaneously, the purple waveform drops significantly and stabilizes, implying that the system has returned to a matched and steady condition.

In Figure 5, the RF generator’s output is shown as a series of periodic on/off RF burst envelopes, each containing a continuous high-frequency sinusoidal waveform. The pulse width and period are clearly visible, demonstrating precise modulation control. This pulse mode effectively reduces the average power while preserving a high peak power. The sharp pulse edges further confirm the fast modulation response and accurate RF switching capability of the system.

In terms of the adaptive frequency sweep algorithm, the following performance characteristics were observed:

1. The RF output waveform clearly exhibits a linear frequency increase from low to high, confirming the LFM nature of the sweep.
2. The signal remains continuous and glitch-free throughout the sweep process, indicating high modulation linearity and excellent control stability.
3. The system completes the adaptive sweep and achieves output stabilization within 5.0172 ms, reflecting fast convergence of the algorithm.
4. The reflected signal remains low in amplitude with no persistent oscillations, signifying good impedance matching and minimal reflected power.

## 6. Conclusions

This experiment successfully validates the output behavior and system response of the RF generator operating in pulse-modulated, self-adaptive sweep mode. The key findings are as follows:

1. The adaptive frequency sweep process is linear and stable. It reflects that the algorithm achieves linear incremental scanning within a predefined frequency range (e.g., 13.0–14.0 MHz), avoids nonlinear fluctuations, ensures precise matching between the source impedance and load impedance, reduces the reflection coefficient, and suppresses pulse transient noise through stability. In plasma excitation, it supports uniform ion distribution and improves the wafer processing yield.
2. The generator exhibits fast startup and produces clean RF output. Fast startup adapts to the pulse “*ON*” time (*T_ON_* = 25 µs), enabling immediate frequency locking and reducing startup latency; it lowers harmonic interference, prevents plasma sheath instability, and avoids etching deviations or deposition defects. In thin film deposition processes, it enhances deposition rates.
3. The system achieves good load matching, with low reflection throughout operation, it suppresses the standing wave ratio, improving continuous operation reliability; in high-aspect-ratio etching, it ensures process uniformity and reduces defect rates.
4. The proposed adaptive sweep algorithm shows a strong application potential in scenarios requiring high precision and reliability, such as RF plasma excitation and impedance matching network testing. High precision supports complex loads (e.g., plasma state transitions) and reliability ensures long-term stable operation, adapting to multi-frequency superposition (13.56 MHz + 2 MHz); it facilitates integration with IMN testing, optimizes network parameters, and reduces manual debugging time. In RF plasma excitation, it enhances system integration and drives innovation in sub−2 nm semiconductor processes.

## Figures and Tables

**Table 1:** Algorithm and relevant parameter settings.

Parameters Name	Parameter Definition	Value
Pulse frequency	**/**	20 kHz
Duty cycle	20 kHz Pulse Duty cycle	50%
Load impedance	Plasma etching chamber impedance	50 Ω
*f_min_*	Start Frequency of Sweep	13.06 MHz
*f_max_*	Start Frequency of Sweep	14.06 MHz
Δ*f_0_*	initial step size	10 KHz
*Γ_threshold_*	reflection coefficient threshold	0.05
*T_ON_*	Pulse “ON” time	25 µs
*f_init_*	Current frequency or Historical best frequency	**/**
*K*	scaling factor	10^4^

## Data Availability

The original contributions presented in the study are included in the article, further inquiries can be directed to the corresponding author.
